# KSHV: Immune Modulation and Immunotherapy

**DOI:** 10.3389/fimmu.2019.03084

**Published:** 2020-02-07

**Authors:** Grant Broussard, Blossom Damania

**Affiliations:** ^1^Lineberger Comprehensive Cancer Center, University of North Carolina at Chapel Hill, Chapel Hill, NC, United States; ^2^Curriculum in Genetics and Molecular Biology, University of North Carolina at Chapel Hill, Chapel Hill, NC, United States; ^3^Department of Microbiology and Immunology, University of North Carolina at Chapel Hill, Chapel Hill, NC, United States

**Keywords:** KSHV, immune system modulation, immunotherapy, oncoviruses, interferon, cytokines

## Abstract

Kaposi's sarcoma (KS)-associated herpesvirus (KSHV) is associated with KS, primary effusion lymphoma (PEL), and multicentric Castleman disease (MCD). To ensure its own survival and propagation, KSHV employs an extensive network of viral proteins to subvert the host immune system, resulting in lifelong latent infection. Modulation of cellular and systemic immune defenses allows KSHV to persist in the host, which may eventually lead to the progression of KSHV-associated cancers. Due to KSHV's reliance on modifying immune responses to efficiently infect its host, immunotherapy is an attractive option for treating KSHV-associated malignancies. In this review, we will focus on the mechanisms by which KSHV evades the immune system and the current immune-related clinical strategies to treat KSHV-associated disease.

## Introduction

The evolutionary arms race between Kaposi's sarcoma (KS)-associated herpesvirus (KSHV, or human herpesvirus 8), an oncogenic gammaherpesvirus, and the immune system of its human hosts has been ongoing for millions of years. The result of this evolutionary competition is the presence of a variety of immunomodulatory proteins within the 165 kB double-stranded DNA viral genome of KSHV. Many of these proteins are pirated orthologs of host immune proteins and enable immune escape (1, 2). The goal of this immunomodulation is to establish viral persistence within the host. KSHV has two phases in its life cycle: latency and lytic replication. During latency, KSHV quiescently persists as a circular episome in host cell nuclei. During the cell cycle, KSHV uses host machinery to replicate its episomes, resulting in the distribution of viral genomes to daughter cells. Following establishment of latency, sporadic bouts of reactivation can lead to the onset of the lytic cycle, which involves transcription of the entire KSHV genome and the production of progeny virions. While lytic replication facilitates transmission of the virus within and between hosts, it increases the risk of the virus being exposed to immune detection (3). Viral proteins present in the latent and lytic life cycle of KSHV have immunomodulatory functions that thwart host antiviral responses. KSHV-associated diseases, such as KS, primary effusion lymphoma (PEL), and multicentric Castleman disease (MCD), often, but not always, occur in patients with immune dysfunction induced by HIV infection or immunosuppressant drugs, indicating the importance of host immunity in restricting viral pathogenesis (4).

Research into interactions between the virus and the host immune system has enabled attempts to clinically reverse immune escape to treat KSHV-associated cancers. This review will summarize how KSHV subverts host intrinsic and cell-mediated immunity, as well as how new immunotherapies are targeting viral immune evasion to treat KSHV-associated disease.

## Modulation of Intrinsic Immunity

**Figure 1:**
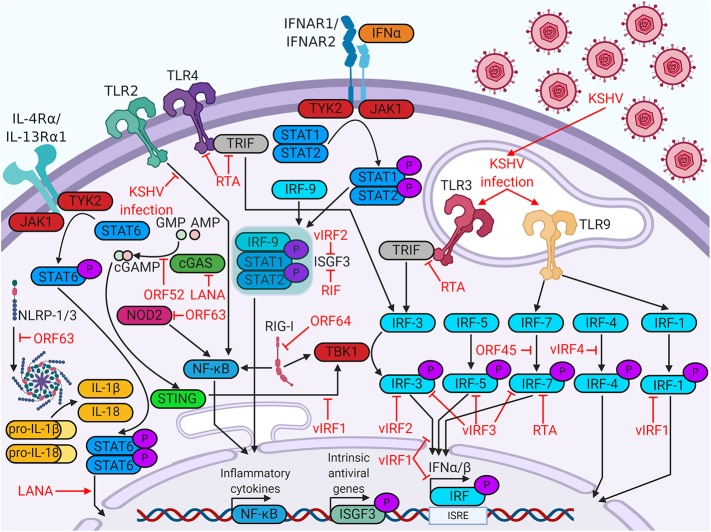
Kaposi's sarcoma (KS)-associated herpesvirus (KSHV) proteins enable evasion of intrinsic immunity. KSHV proteins antagonize viral sensing and interferon responses by targeting a wide variety of host immune factors.

KSHV targets many steps of the host immune response, beginning with viral detection. Cells detect viruses through the binding of pathogen-associated molecular patterns (PAMPs) to pattern recognition receptors (PRRs). PRR stimulation activates signaling pathways that promote transcription of antiviral genes, interferon production, or apoptosis (5). In the following sections, we will discuss how KSHV impacts the intrinsic immune response by modulating PRRs and altering interferon production in infected cells (Figure 1).

The most well-understood PRRs are the Toll-like receptors (TLRs). Six human TLRs are localized to the plasma membrane (TLR1/2/4/5/6/10), whereas the remaining four are expressed in intracellular vesicles (TLR3/7/8/9). TLRs in various cellular compartments recognize different PAMPs; membrane TLRs recognize structural components of invasive pathogens, such as viral glycoproteins, whereas vesicular TLRs recognize pathogenic DNA or RNA (6). In monocytes, KSHV infection upregulates transcripts for TLR3 and its downstream targets, interferon β (IFNβ), C-C motif chemokine ligand 2 (CCL2), and C-X-C motif chemokine 10 (CXCL10) (7). The KSHV viral interferon regulatory factors (vIRFs), however, reduce TLR3-mediated interferon induction (8). Another KSHV protein that regulates TLR signaling is replication and transcription activator (RTA) protein. RTA promotes proteasomal degradation of TLR3 adaptor protein TRIF, blocking downstream signaling (9). RTA also blocks TLR4 signaling by promoting degradation of mRNA encoding adaptor protein myeloid differentiation primary response protein 88 (MyD88) (10). KSHV infection in endothelial cells rapidly suppresses TLR4 signaling *via* viral G-protein-coupled receptor (vGPCR)- and vIRF1-mediated activation of extracellular signal-regulated kinase (ERK) signaling (11). KSHV suppression of TLR2 signaling has been demonstrated in infected THP-1 monocytes, potentially due to RTA-mediated downregulation of TLR2 protein expression (12). KSHV infection in plasmacytoid dendritic cells activates TLR9 signaling, leading to increased CD83, CD86, and IFNα expression (13). The intricate relationships between KSHV and TLRs highlight the importance of these host factors in viral restriction.

Although TLRs are the most studied PRRs, KSHV antagonizes a variety of other PRRs. Nucleotide-binding oligomerization domain-like receptors, or NOD-like receptors (NLRs), are cytosolic sensors of PAMPs. Some activated NLRs form complexes known as inflammasomes, which recruit apoptotic caspases and promote production of inflammatory cytokines, such as IL-1β or IL-18 (6). KSHV open reading frame 63 (ORF63) binds several NLR family members, including NLRP1, thereby interfering with inflammasome formation and inflammatory cytokine production (14). Retinoic acid-inducible gene I (RIG-I)-like receptors (RLRs) canonically activate and upregulate type I interferon in response to double-stranded RNA and were implicated in sensing DNA viruses as well (6, 15). Viral deubiquitinase ORF64 suppresses RIG-I by preventing its ubiquitination and activation (16). KSHV also antagonizes cytosolic DNA sensors, such as cyclic GMP-AMP synthase (cGAS). The cyclic GMP-AMP (cGAMP) produced by cGAS binds and activates stimulator of interferon genes (STING) on the ER membrane. Activated STING recruits interferon-inducing proteins, such as TANK binding kinase 1 (TBK1) and interferon regulatory factor 3 (IRF3). KSHV targets the cGAS-STING pathway at multiple points. KSHV ORF52 binds to and inhibits the enzymatic activity of cGAS, blocking the production of cGAMP (17). LANA also binds cGAS, blocking downstream activation of TBK1 (18). vIRF1 binds directly to STING and blocks the recruitment of TBK1 (19). Clearly, evasion of a wide variety of cellular viral sensors is critical for maintenance of persistent KSHV infection.

Host cell identification of viral pathogens can trigger an interferon response. Interferons are a class of cytokines which activate immune responses against pathogens. There are three major types of interferon- type I, II, and III. Type II interferon solely includes IFNγ, which plays important roles in responses against non-viral pathogens. Type I interferon (IFNα/βs) and type III interferon (IFNλs) engage their receptors to induce antiviral pathways in infected cells and their neighbors (20). In this antiviral state, cells restrict viral replication, increase expression of major histocompatibility complex class I (MHC-I), and are more likely to undergo growth arrest or apoptosis. The production of these cytokines is induced by the phosphorylation, dimerization, and nuclear translocation of interferon regulatory factors, which are transcription factors that recognize interferon gene promoters (21). The KSHV genome encodes four viral homologs of cellular IRFs (vIRF1 to 4), all of which are pirated from the host genome. The vIRFs are known to target multiple cellular proteins, including cellular IRFs, to inhibit interferon responses [reviewed in (22)].

vIRFs are not the only KSHV proteins that target the interferon response. ORF45 prevents phosphorylation and activation of IRF7 by competing with its kinases, IκB kinase ε (IKKε) and TBK1, as an alternative substrate (23). RTA promotes the proteasomal degradation of IRF7 by acting as an E3 ubiquitin ligase (24). KSHV Latency Associated Nuclear Antigen (LANA) and basic-region leucine zipper protein (K-bZIP) bind to the IFNβ promoter, inhibiting transcriptional activity and blocking the interferon response (25, 26). Some viral proteins, such as regulator of interferon production (RIF), block type I interferon further downstream, complexing with ISGF3 subunits to prevent complex formation and subsequent antiviral transcriptional activity (27).

## Modulation of Cell-Mediated Immunity

**Figure 2:**
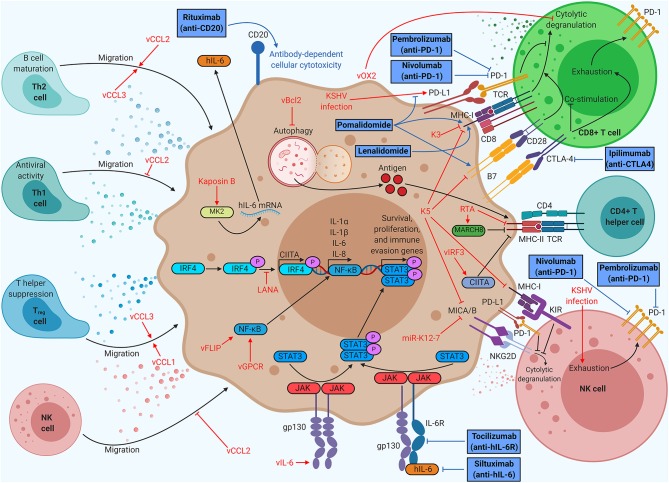
Kaposi's sarcoma (KS)-associated herpesvirus (KSHV) proteins enable evasion of cell-mediated immunity, but immunotherapy can restore function. KSHV proteins recruit beneficial helper cell populations, create an inflammatory microenvironment, and interfere with T and NK cell function. Therapies targeting antigen presentation or immune checkpoints provide durable clinical benefit in KSHV-associated malignancies. Anti-PD1 therapy (nivolumab/pembrolizumab) has already demonstrated clinical benefits for KS patients, spurring ongoing clinical trials of anti-PD1 monotherapy and dual anti-PD1/anti-CTLA4 (ipilimumab) therapy. Thalidomide analogs (pomalidomide and lenalidomide) have been shown to increase CD8^+^ T cell killing by modulating PD-L1, MHC-I, and B7 expression, prompting KS clinical trials of monotherapy and dual therapy with liposomal doxorubicin. Thalidomide analogs are being explored in combination with chemotherapy and rituximab for the treatment of PEL in clinical trials. Anti-IL-6 (siltuximab) has shown efficacy for idiopathic multicentric Castleman disease (MCD), prompting clinical trials of anti-IL-6R (tocilizumab) for KSHV-MCD.

In addition to subverting the intrinsic defenses of host cells, KSHV also subverts the immune system of the entire human host (Figure 2). Immune cells, such as dendritic cells, macrophages, natural killer (NK) cells, and T cells, survey the host for pathogens. The number, activity, localization, and differentiation state of these cells are regulated by cytokine signaling. Pro-inflammatory cytokines typically increase immune cell number and function, whereas anti-inflammatory cytokines stifle these responses. Immune cell localization is controlled by chemokines, which are cytokines that recruit specific cell types to sites of immunological activity.

Virally infected cells are recognized and destroyed by two cell types: CD8^+^ T cells and NK cells. CD8^+^ T cells bind viral antigens displayed on MHC-I by infected cells, leading to secretion of cytokines, release of cytotoxic granules, or Fas-mediated apoptosis of the target cell (28). If the target cell has compromised MHC-I expression, this process cannot occur. NK cells cover this gap in immune defense, releasing cytotoxic granules targeting any cell they encounter that does not express MHC-I. To become fully activated, NK cells require binding of stress markers like MHC-I polypeptide-related sequence A or B (MICA/B) to the NK cell receptor, natural killer group 2D (NKG2D) receptor (2). Although not directly cytotoxic, CD4^+^ T helper cells also play a role in viral pathology. CD4^+^ T helper cells recognize exogenous antigens displayed on MHC-II by phagocytic cells. Each subclass of CD4^+^ T cells plays a different role in immune function. Type 1 T helper (Th1) cells typically produce inflammatory responses, spurring intrinsic immunity, whereas activated Th2 cells promote the differentiation of B cells, spurring humoral immunity through antibody production. Regulatory T (T_reg_) cells secrete anti-inflammatory cytokines and suppress helper T cell function, restricting cell-mediated immunity (29).

### Modulation of Cytokine Signaling

KSHV induces an inflammatory cytokine milieu, particularly when the host has developed a KSHV-associated malignancy, which enables growth and proliferation of infected cells while crippling certain aspects of the host immune system. Primary KSHV infection in monocytes induces secretion of IL-1α, IL-1β, and IL-6, all of which are pro-inflammatory (30). The sera of KS patients show high levels of inflammatory cytokines like IL-6 and TNFα, as well as elevated IL-10, an immunosuppressive cytokine (31). Chronic inflammation is a hallmark of KSHV-associated disease. Both MCD and a recently defined inflammatory syndrome, KSHV-associated inflammatory cytokine syndrome (KICS), are indicated by very high levels of IL-6 and IL-10 in the sera (32, 33).

KSHV encodes a viral homolog of IL-6 (vIL-6). Whereas IL-6 requires IL-6R and gp130 for signaling, vIL-6 signals through gp130 dimers (34). Both cytokines induce Janus kinase (JAK)-STAT signaling, particularly through STAT3, which is associated with cell proliferation, angiogenesis, migration, and differentiation (35–37). vIL-6 also stimulates increased secretion of IL-6, resulting in KS/PEL progression or MCD flare-ups due to heightened B cell proliferation (38). Low levels of latent vIL-6 expression in PEL cells contribute to cellular proliferation and survival (39). vIL-6 has been shown to upregulate activation-induced cytidine deaminase (AID) expression in stimulated B cells, leading to increased class-switch recombination (40). Although it is unclear how this might influence the pathology of PEL or MCD, it is clear that vIL-6 induces major changes within the host immune system. Other viral factors contribute to the dramatic upregulation of IL-6 associated with KSHV-related disease. Kaposin B stabilizes IL-6 mRNA *via* binding and activation of MAPK-activated protein kinase 2 (MK2) (41). Both viral FLICE-inhibitory protein (vFLIP) and vGPCR (an IL-8R homolog) activate the NF-κB pathway, a pro-inflammatory pathway that increases secretion of cytokines like IL-6 and IL-8 (42). vFLIP binds the regulatory IκB kinase γ (IKKγ) protein, promoting NFκB nuclear translocation and transcriptional activation (43). vGPCR induces Rac family small GTPase 1 (Rac1) signaling, which also activates NF-κB (44). KSHV also modulates IL-4/13-STAT6 signaling by inducing constitutive phosphorylation of STAT6. LANA-mediated cleavage and subsequent nuclear translocation of STAT6 generate a transcriptional repressor of RTA. This hijacked signaling pathway suppresses viral reactivation from latency, enabling evasion of immune surveillance (45–49) (Figure 1).

KSHV has three viral homologs of cellular chemokines, vCCL1 to vCCL3 (also known as vMIP-I to vMIP-III). vCCL2 binds to a wide variety of chemokine receptors on many different cell types and can have agonistic or inhibitory effects. vCCL1 binds to CCR8, which is prominently expressed on T_reg_ cells, suggesting that the virus may recruit this T cell subtype (50). vCCL2 has been shown to bind CX3C chemokine receptor 1 (CX3CR1) and CCR5, blocking their natural ligands and inhibiting the migration of naive and activated NK cells (51). vCCL2 antagonizes CCR1 and CCR5 activation, which are primarily found on Th1 cells, while stimulating CCR3 and CCR8, which has been shown to attract Th2 cells to KS lesions (52). Th1 cells are more prominently antiviral than Th2 cells, so this protein may allow immune escape of infected cells. vCCL3 is an agonist of CCR4, another chemokine receptor found preferentially on Th2 and T_reg_ cells. vCCL3 also preferentially induces chemotaxis in Th2 cells when compared to Th1 cells, suggesting that viral chemokines may play a role in creating the characteristically Th2-skewed KS microenvironment (52).

### Evasion of Immune Cell Detection

Many KSHV proteins subvert T cell- or NK cell-mediated killing. KSHV K3 is a viral ubiquitin ligase, which promotes degradation of MHC-I, CD1d, CD31, and IFN-γR1, hindering CD8^+^ T cell activation and recognition of infected cells. KSHV K5, another viral ubiquitin ligase, promotes MHC-I, CD54, B7-2, CD1d, MICA, and MICB degradation, interfering with proper CD8^+^ T cell and NK cell function (53). A KSHV microRNA, miR-K12-7, also downregulates MICB expression (54). Viral orexin receptor 2 (vOX2), a viral glycoprotein displayed on the plasma membrane, suppressed interferon production and cytolytic granule mobilization by CD8^+^ T cells when expressed on antigen-presenting cells in coculture (55).

KSHV also attempts to evade CD4^+^ T cell-mediated immunity. KSHV RTA promotes proteasomal degradation of MHC-II while enhancing expression of membrane-associated RING-CH (MARCH8), a MHC-II antagonist (56). MHC-II antigen display requires autophagy, which viral B-cell leukemia/lymphoma 2 (vBcl2) blocks (57). vIRF3 downregulates IFNγ and class II MHC transactivator (CIITA), resulting in lower MHC-II presentation (58). KSHV LANA binds to and blocks IRF4 from activating the CIITA promoter, resulting in decreased MHC-II expression (59).

## Immunotherapy For Kshv-Related Diseases

KSHV's extensive modulation of the host immune system allows many opportunities for clinical intervention. The utility of this concept has been validated by the success of antiretroviral drug therapy (ART)-induced immune reconstitution in treating early KS, inducing regression in 80% of patients living with HIV (60). Immune responses require recognition of target cells, adequate costimulation, and the absence of inhibitory markers. Unfortunately, in many disease states, target cells will overexpress an inhibitory marker, leading to suppression of an effective immune response. Furthermore, chronic stimulation of immune cells can lead to an “exhausted” phenotype, where cells overexpress inhibitory markers and become unable to respond to stimulation. Immunotherapy attempts to restore or boost the function of immune cells, primarily through antibodies directed at various immune receptors.

One of the most successful immunotherapies to date is anti-programmed cell death protein 1 (PD-1)/programmed death ligand 1 (PD-L1) therapy. PD-L1 is an inhibitory molecule that is overexpressed on many tumor types, enabling immune escape. KSHV infection leads to increased PD-L1 expression in monocytes and could contribute to immune evasion (30). NK cells isolated from KS patients consistently show elevated levels of PD-1, indicative of an exhausted phenotype (61). Anti-PD-1 antibodies (nivolumab or pembrolizumab) demonstrated significant antitumor effects in a small group of patients with HIV-associated KS (62). An ongoing phase I clinical trial (ClinicalTrials.gov Identifier: NCT03316274) aims to treat patients with limited cutaneous KS with intralesional injections of nivolumab. Pembrolizumab has demonstrated safety and clinical benefits in patients living with HIV and KS as part of a phase I clinical trial (NCT02595866) and is currently being used in a phase II trial for the treatment of KS (NCT03469804) (63). Anti-cytotoxic T-lymphocyte-associated protein 4 (CTLA-4) is another common target for immunotherapy due to its role in suppressing B7-mediated costimulation of T cells (64). There is an ongoing phase II clinical trial testing a combinatorial therapy of nivolumab and ipilimumab, an anti-CTLA-4 antibody, on classical KS patients (NCT03219671). Targeting two separate T cell inhibitory proteins at once may result in greater T cell function.

Pomalidomide, a thalidomide analog, has recently been granted breakthrough status by the FDA for the treatment of KS based on an ongoing phase I/II clinical trial (NCT01495598), prompting the design of a similar phase II clinical trial in a sub-Saharan Africa (NCT03601806) (65). Pomalidomide was shown to restore MHC-I expression in the context of lytic replication, as well as restoring intercellular adhesion molecule 1 (ICAM-1) and B7-2 levels in latently infected PEL cells (66). Pomalidomide has also been shown to inhibit PD-L1 upregulation by tumor cells, leading to increased CD8^+^ T cell killing (67). Clinical trials for pomalidomide with liposomal doxorubicin in advanced or refractory KS (phase I, NCT02659930) and pomalidomide monotherapy for KS in a sub-Saharan Africa cohort (phase II, NCT03601806) are currently in progress.

Unlike KS, PEL and MCD are B cell proliferative diseases. Rituximab, an anti-CD20 antibody, which induces B cell death upon binding, has shown efficacy in the treatment of KSHV-MCD despite a common lack of CD20 expression on these plasmablasts (68). Similarly, PEL cells usually do not express CD20, but rituximab may still provide clinical benefit (69). Rituximab treatment decreases levels of IL-6, potentially through elimination of CD20^+^ cytokine-secreting B cells. High IL-6 levels correlate with poor prognosis in PEL and drive progression of MCD (70). A phase I/II clinical trial of lenalidomide, another thalidomide analog, combined with chemotherapy (DA-EPOCH) and rituximab (NCT01495598), is currently ongoing. Lenalidomide has been shown to inhibit lytic MHC-I downregulation induced by KSHV K3 (66). Since MCD is characterized by high levels of cellular IL-6, anti-IL-6 antibodies have been considered for new therapies. Idiopathic MCD, which is thought to arise due to cellular IL-6 overexpression, responds well to siltuximab, an anti-IL-6 antibody (71). However, KSHV-MCD arises due to combined effects of IL-6 and vIL-6, and IL-6 targeting antibodies will not directly bind to vIL-6. A phase II clinical trial of tocilizumab, an anti-IL-6R antibody, in KSHV-MCD (NCT01441063) is currently underway.

## Concluding Remarks

KSHV has evolved with the human immune system for millions of years and has developed mechanisms to antagonize it at many steps: viral detection, interferon signaling, cytokine production, and immune cell recruitment and function. However, the reliance of this virus on its immunomodulatory properties offers a window for clinical intervention. As our understanding of the immune system expands, new therapeutic targets will arise. Given the success of immunotherapy in treating KSHV-associated malignancies, the discovery of novel immune checkpoints has the potential to translate into clinical benefits for patients with these diseases. Additionally, just as the virus has pirated genes from the human genome, we too may learn from the virus how to effectively modulate immune responses. This kind of information could be applied to the treatment of autoimmune diseases, where immunosuppression is required to prevent disease progression. Although there are many aspects of KSHV immunomodulation that are unclear, the discoveries that have been made thus far have built the foundation for the breakthrough therapies we benefit from today.

## Author Contributions

GB and BD wrote and edited this review.

### Conflict of Interest

The authors declare that the research was conducted in the absence of any commercial or financial relationships that could be construed as a potential conflict of interest.
